# Diagnostic utility of plasma translocator protein 18 kDa (TSPO) in sepsis: A case–control study

**DOI:** 10.1097/MD.0000000000040396

**Published:** 2024-11-01

**Authors:** Miyuki Hattori, Kazuya Kikutani, Koji Hosokawa, Michihito Kyo, Mitsuaki Nishikimi, Kohei Ota, Shinichiro Ohshimo, Hidenori Aizawa, Nobuaki Shime

**Affiliations:** a Department of Emergency and Critical Care Medicine, Graduate School of Biomedical and Health Sciences, Hiroshima University, Hiroshima, Japan; b Department of Neurobiology, Graduate School of Biomedical and Health Sciences, Hiroshima University, Hiroshima, Japan; c Department of Anesthesiology and Reanimatology, Faculty of Medicine Sciences, University of Fukui, Fukui, Japan; d Department of Radiation Disaster Medicine, Research Institute for Radiation Biology and Medicine, Hiroshima University, Hiroshima, Japan.

**Keywords:** biomarker, sepsis, translocator protein

## Abstract

Translocator protein 18 kDa (TSPO) is a mitochondrial membrane protein that is involved in inflammation, oxidative stress, and steroidogenesis. TSPO may be a marker of inflammatory responses in the brain and other organs, but there have been few studies of the potential clinical significance of measuring the circulating TSPO concentration, especially in patients with sepsis. In this study, we compared the circulating TSPO concentrations of patients with sepsis and healthy controls to investigate the utility of plasma TSPO for the diagnosis of sepsis. Patients with sepsis admitted to the intensive care unit of Hiroshima University Hospital between January 2020 and April 2024 were enrolled. Plasma samples were collected from patients within 24 hours of admission and also from healthy volunteers, and their plasma TSPO concentrations were compared. Receiver operating characteristic analysis was used to evaluate the usefulness of plasma TSPO concentration for the diagnosis of sepsis. We also investigated the relationships of TSPO concentration with the severity of sepsis, complications, and prognosis of the patients. Eighty subjects (52 patients and 28 controls) were included in this study. The plasma TSPO concentrations of the patients with sepsis were significantly lower than those of the healthy controls (0.094 vs 0.25 ng/mL, *P* < .001), and receiver operating characteristic analysis generated an area under the curve of 0.81 (95% confidence interval: 0.72–0.91). In patients with sepsis, the TSPO concentration was not associated with the severity of sepsis, complications, or prognosis. Plasma TSPO may be a useful biomarker for the diagnosis of sepsis.

## 1. Introduction

Translocator protein 18 kDa (TSPO) is a mitochondrial membrane protein that is involved in inflammation, oxidative stress, and steroidogenesis.^[1,2]^ It is well-known for its expression in microglia of the brain, but it is also present in various organs throughout the body.^[3]^ In normal brain tissue, TSPO expression is low, but it significantly increases in response to brain injury or inflammation.^[3]^ Given that it is preferentially expressed in activated microglia and macrophages, it is understood to be related to neuroinflammation associated with such as Alzheimer diseases and Parkinson diseases.^[2,4]^ Furthermore, the development of radioligands targeting TSPO has led to its use in PET-based imaging.^[5,6]^

The role of TSPO in sepsis has also been identified. In a mouse model of sepsis induced by the administration of lipopolysaccharide (LPS), TSPO expression increased in hippocampal microglia, and the administration of a TSPO antagonist ameliorated the associated behavioral abnormalities.^[7]^ In addition, in another study of a mouse model of sepsis induced by cecal ligation and puncture, mice with TSPO deletion exhibited prolonged weight loss compared with wild-type mice, and during the recovery phase, significant depressive- and anxiety-like behaviors, as well as muscle weakness, were observed.^[8]^

An imbalance between the populations of M1 and M2 macrophages is believed to trigger the onset and facilitate the progression of sepsis.^[9]^ During the acute phase of sepsis, M1 macrophages predominate and release significant amounts of proinflammatory mediators, leading to tissue damage.^[9]^ Recent studies have also shown differences in TSPO expression between human M1 and M2 macrophages.^[10]^ However, although changes in TSPO expression have been implicated in sepsis, the potential utility of measuring the plasma TSPO concentrations of patients with sepsis has not been evaluated. Therefore, the aim of the present study was to compare the circulating TSPO concentrations of patients with sepsis with those of healthy controls, and to evaluate the potential clinical utility of TSPO for the diagnosis of sepsis.

## 2. Materials and methods

### 2.1. Patients and sample collection

Patients admitted to the intensive care unit (ICU) at Hiroshima University Hospital because of sepsis between January 2020 and April 2024 were included in the study. Plasma samples were collected from the patients with sepsis within 24 hours of admission. One reason for collecting blood samples within the first 24 hours of admission is the need for early diagnosis in sepsis. The second reason is that previous studies have suggested that changes in TSPO expression of macrophage and peripheral organ occur within a few hours to 24 hours following LPS stimulation.^[10,11]^ We diagnosed sepsis and septic shock using the Sepsis–3 criteria.^[12]^ Here, sepsis was diagnosed in a patient suspected of having an infectious disease, with an increase of 2 points or more in the sequential organ failure assessment (SOFA) score.^[13]^ Furthermore, septic shock was defined as a condition requiring vasopressors to maintain a mean arterial pressure of 65 mm Hg or higher despite adequate fluid resuscitation. Sample collection was performed after obtaining the written consent of the patients or their families, and of the healthy volunteers. The collected samples were stored in a -80 °C freezer until analysis. We collected the following information from the patients: age, sex, site of infection, SOFA score, acute physiology, and chronic health evaluation (APACHE) II score, presence/absence of disseminated intravascular coagulation (DIC), acute respiratory distress syndrome (ARDS), bacteremia, and the outcome at discharge from the ICU. DIC was diagnosed using the Japanese Association for Acute Medicine DIC diagnostic criteria.^[14]^ A diagnosis of ARDS was made on the basis of the Berlin definition.^[15]^ The study was approved by the Ethics Review Committee of Hiroshima University (number E2016-0447-07).

### 2.2 . Measurement of the plasma concentration of TSPO

The plasma TSPO concentration was measured using a commercially available ELISA kit (Wuhan Fine Biotech, EH4447 Human TSPO ELISA Kit, Wuhan, China), largely according to the manufacturer’s instructions. The only difference from these was that while they recommend at least 2-fold dilution of the samples, we did not do this, because preliminary testing indicated low TSPO concentrations. If the concentration was less than the detection sensitivity of the ELISA kit (0.094 ng/mL), the measured value was recorded as 0.094 ng/mL.

### 2.3. Statistical analysis

Continuous data are summarized as median (interquartile range) and categorical data as number (percentage). The comparison of the plasma TSPO concentrations of the groups was performed using the Mann–Whitney *U* test. To evaluate the discriminative ability of serum TSPO concentration for sepsis, we calculated the area under the receiver operating characteristic (ROC) curve. To evaluate the relationships of TSPO concentration with the SOFA score and the APACHE II score, we used the Spearman rank correlation coefficient. We set the significance level at *P* < .05. All statistical analyses were conducted using EZR (Saitama Medical Center, Jichi Medical University, Saitama, Japan).^[16]^

## 3. Results

### 3.1. Patient characteristics

A total of 80 subjects (52 patients with sepsis and 28 healthy controls) were included in the study. Patient flow is shown in Figure S1, Supplemental Digital Content, http://links.lww.com/MD/N858. The characteristics of the patients are shown in Table 1. The median age of the patients with sepsis was 74.0 years (interquartile range 60.0–81.0 years), and 38 (73.0%) were male. Their median SOFA score was 9.5 (7.0–12.0) and their median APACHE II score was 23.5 (19.0–28.3). The sites of infection were the abdomen in 19 (36.5%) patients, the urinary tract in 12 (23.1%), a lung in 10 (19.2%), soft tissue in 7 (13.5%), and others in 4 (7.7%). Four patients died in the ICU (7.7%).

**Table 1:** Patient characteristics.

	Sepsis (N = 52)	Healthy control (N = 28)
Age	74.0 [60.0, 81.0]	34.5 [28.0, 45.5]
Sex (male)	38 (73.1)	16 (57.1)
SOFA	9.5 (7.0–12.0)	
APACHEII	23.5 (19.0–28.3)	
Septic shock	45 (86.5)	
ARDS	11 (21.2)	
DIC	33 (63.5)	
Bacteremia	28 (60.9)	
Infection focus		
Abdomen	19 (36.5)	
Urinary tract	12 (23.1)	
Lung	10 (19.2)	
Soft tissue	7 (13.5)	
Others, unknown	4 (7.7)	
ICU mortality	4 (7.7)	

Values presented as median [interquartile range] or number (percent).

APACHEII = acute physiology and chronic health evaluation II score, ARDS = acute respiratory distress syndrome, DIC = disseminated intravascular coagulation, ICU = intensive care unit, SOFA = sequential organ failure assessment.

The healthy controls had a median age of 34.5 years (28.0–45.5 years) and 16 (57.1%) were male.

### 3.2 . Patients with sepsis have significantly lower plasma TSPO concentrations than healthy controls

**Figure 1.**
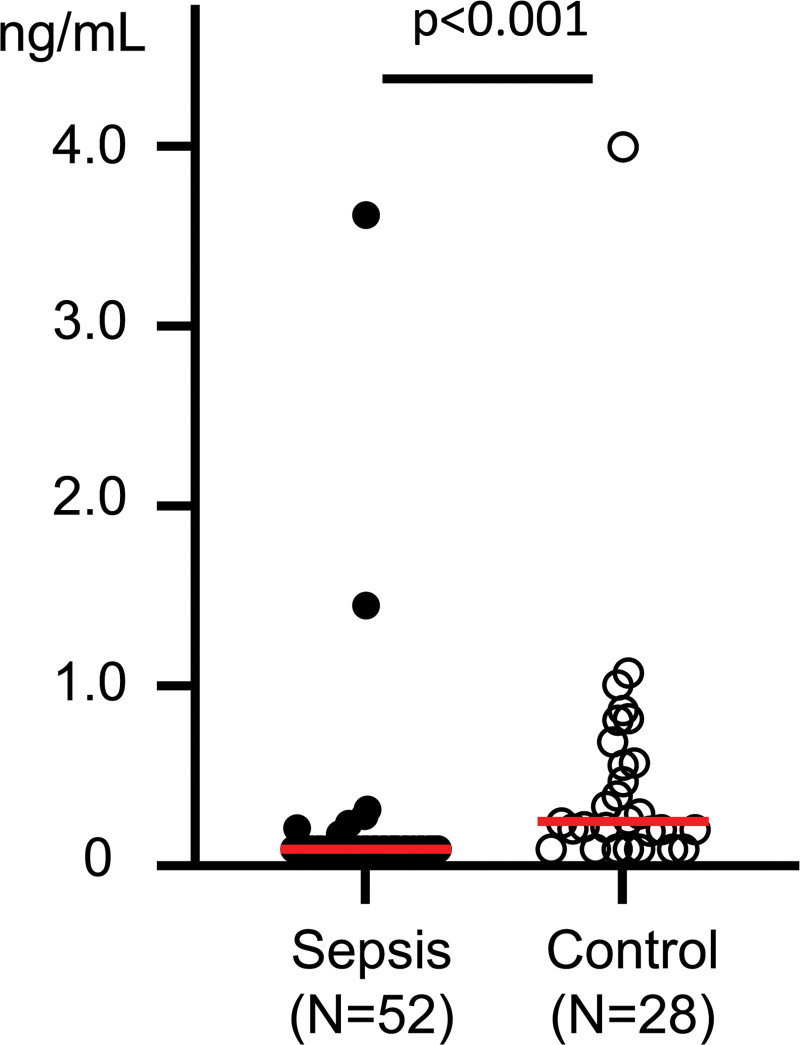
Comparison of the plasma TSPO concentrations of patients with sepsis and healthy controls. Patients with sepsis (N = 52) had significantly lower plasma TSPO concentrations than healthy controls (N = 28) (Mann–Whitney *U* test, 0.094 vs 0.25 ng/mL, *P* < .001). The red line indicates the median for each group. TSPO = translocator protein 18 kDa.

**Figure 2.**
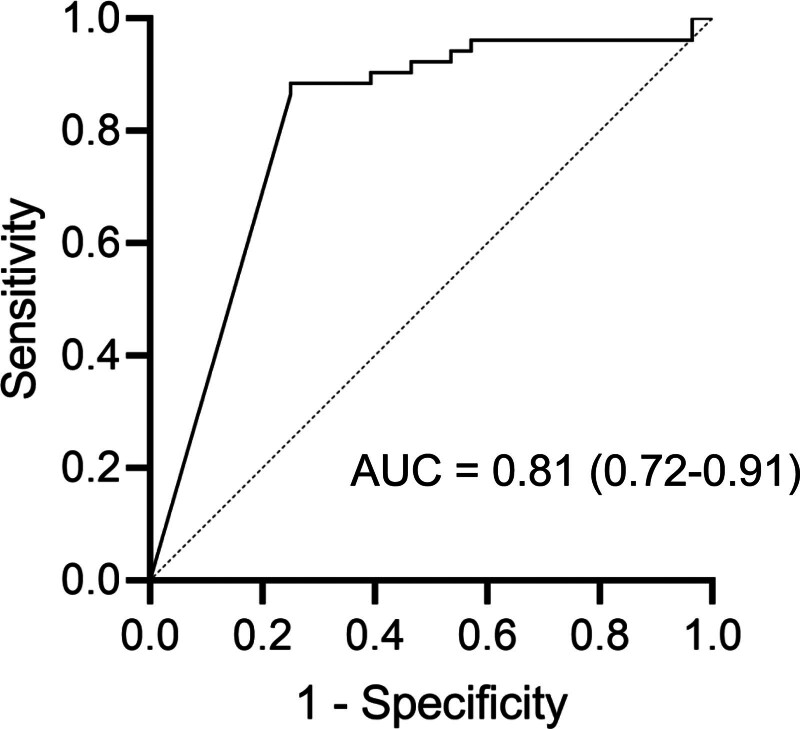
Receiver operating characteristic (ROC) curve analysis of the use of plasma TSPO concentration for the diagnosis of sepsis. ROC analysis showed that the area under the curve (AUC) for the diagnosis of sepsis was 0.81 (95% confidence interval: 0.72–0.91).

The plasma TSPO concentrations were below the detection sensitivity in 45 (87%) of the patients with sepsis and in 7 (25%) of the healthy controls. The plasma TSPO concentrations of the patients with sepsis were significantly lower than those of healthy controls (0.094 vs 0.25 ng/mL, *P* < .001) (Fig. 1). ROC analysis generated an area under the curve for the identification of individuals with sepsis of 0.81 (95% confidence interval: 0.72–0.91) (Fig. 2). The cut off value where sensitivity + specificity is maximized was 0.18 ng/mL, with sensitivity and specificity values of 0.75 and 0.89, respectively.

### 3.3. Relationships between the severity of sepsis, complications, and outcomes with the TSPO concentration

Significant correlations were not obtained for the relationships of TSPO concentration with the SOFA score (*R* = 0.26, *P* = .06) or the APACHE II score (*R* = 0.06, *P* = .68). There was no significant difference in the TSPO concentrations of patients who died in the ICU and those who were discharged alive (0.094 vs 0.094, *P* = .44). There were also no significant relationships of the TSPO concentration with the presence of complications of sepsis (septic shock (*P* = .28), ARDS (*P* = .65), DIC (*P* = .22), and bacteremia (*P* = .27)).

## 4. Discussion

In the present study, we have demonstrated that the plasma TSPO concentrations of patients with sepsis admitted to the ICU are significantly lower than those of healthy controls. Furthermore, ROC analysis suggested the potential utility of TSPO as a useful marker for the diagnosis of sepsis. To the best of our knowledge, this is the first study to assess the plasma TSPO concentrations of patients with sepsis.

Few previous studies have involved the quantification of the circulating TSPO concentrations of humans. In patients with acute ischemic stroke^[17]^ and spontaneous intracerebral hematoma,^[18]^ high circulating TSPO concentrations have been shown to be associated with poor functional outcomes. In addition, in older women with breast cancer, a high serum TSPO concentration has been shown to be significantly associated with the onset of postoperative delirium.^[19]^ Contrary to previous studies, which showed that high TSPO concentrations are associated with worse clinical outcomes, in the present study we have shown that the plasma TSPO concentrations of patients with sepsis are low. In a previous study of a mouse model of sepsis, induced using LPS, an upregulation of TSPO was identified specifically in activated microglia.^[7]^ However, this high expression of TSPO was not identified in human microglia or macrophages.^[20]^ It has been suggested that this may be explained by differences in the promoter regions of the TSPO gene in rodents and humans.^[21]^ Furthermore, recent studies of human peripheral blood have shown that TSPO is downregulated as part of the M1 polarization of macrophages.^[10]^ It is known that in the acute phase of sepsis, such as in the patients we studied, M1 differentiation predominates.^[9]^ Therefore, it is likely that the macrophage-related inflammatory response contributes to the low plasma TSPO concentrations of patients with sepsis. In future studies, it will be necessary to investigate how TSPO expression changes during the acute and chronic phases of sepsis progression across various tissues.

There were several limitations to the present study. First, because the samples were obtained within 24 hours of ICU admission and the time course from the onset of sepsis was not standardized, it could not be determined at what stage of the immune response to sepsis the measurements were made. Second, because TSPO is a peripheral benzodiazepine receptor, the measured concentrations may have been affected by the use of anesthetic agents associated with tracheal intubation or other medication. Third, the age of the patients with sepsis and healthy controls in the present study differed greatly, and therefore age may have affected the TSPO concentrations of the patients. We observed the possibility that, in the analysis of 28 healthy controls, age is positively correlated with plasma TSPO concentration, but the sample size was small and the study power was lacking. Finally, the TSPO concentration could only be measured in 7 patients with sepsis (13.4%), which may have been responsible for the weak associations between TSPO and clinical outcomes. We need validation through techniques such as protein concentration, development of more sensitive kits, and measurement using mass spectrometry. However, despite these limitations, TSPO may be useful for the diagnosis of sepsis, and this possibility warrants further investigation.

## 5. Conclusions

Our study observes that plasma TSPO concentrations are significantly lower in patients with sepsis compared to healthy controls. However, several limitations should be noted. The variability in sample collection timing, potential influence of medications, age differences between groups, and the small subset of measurable TSPO levels in sepsis patients could affect the results. Even considering these limitations, low plasma TSPO concentration in sepsis suggested a possible diagnostic marker for sepsis. Further research is needed to confirm its clinical utility.

## Acknowledgments

We thank Mark Cleasby, PhD from Edanz (https://jp.edanz.com/ac) for editing a draft of this manuscript.

## Author contributions

**Conceptualization:** Miyuki Hattori, Kazuya Kikutani.

**Data curation:** Miyuki Hattori, Kazuya Kikutani, Michihito Kyo, Mitsuaki Nishikimi.

**Formal analysis:** Miyuki Hattori, Kazuya Kikutani.

**Funding acquisition:** Kazuya Kikutani, Kohei Ota, Nobuaki Shime.

**Investigation:** Miyuki Hattori, Kazuya Kikutani.

**Methodology:** Miyuki Hattori, Kazuya Kikutani.

**Project administration:** Kazuya Kikutani.

**Resources:** Miyuki Hattori, Kazuya Kikutani, Hidenori Aizawa.

**Supervision:** Hidenori Aizawa, Nobuaki Shime.

**Visualization:** Miyuki Hattori, Kazuya Kikutani.

**Writing – original draft:** Miyuki Hattori, Kazuya Kikutani.

**Writing – review & editing:** Koji Hosokawa, Michihito Kyo, Mitsuaki Nishikimi, Kohei Ota, Shinichiro Ohshimo, Hidenori Aizawa, Nobuaki Shime.

## Supplementary Material


