# Supplementary material for: Diagnostic utility of plasma translocator protein 18 kDa (TSPO) in sepsis: A case–control study
Source: Medicine (Baltimore). 2024 Nov 1;103(44):e40396. doi: 10.1097/MD.0000000000040396 (PMC11537663; doi:10.1097/MD.0000000000040396)
Supplement: Supplementary file 1 [file medi-103-e40396-s001.pptx]

## Slide 1
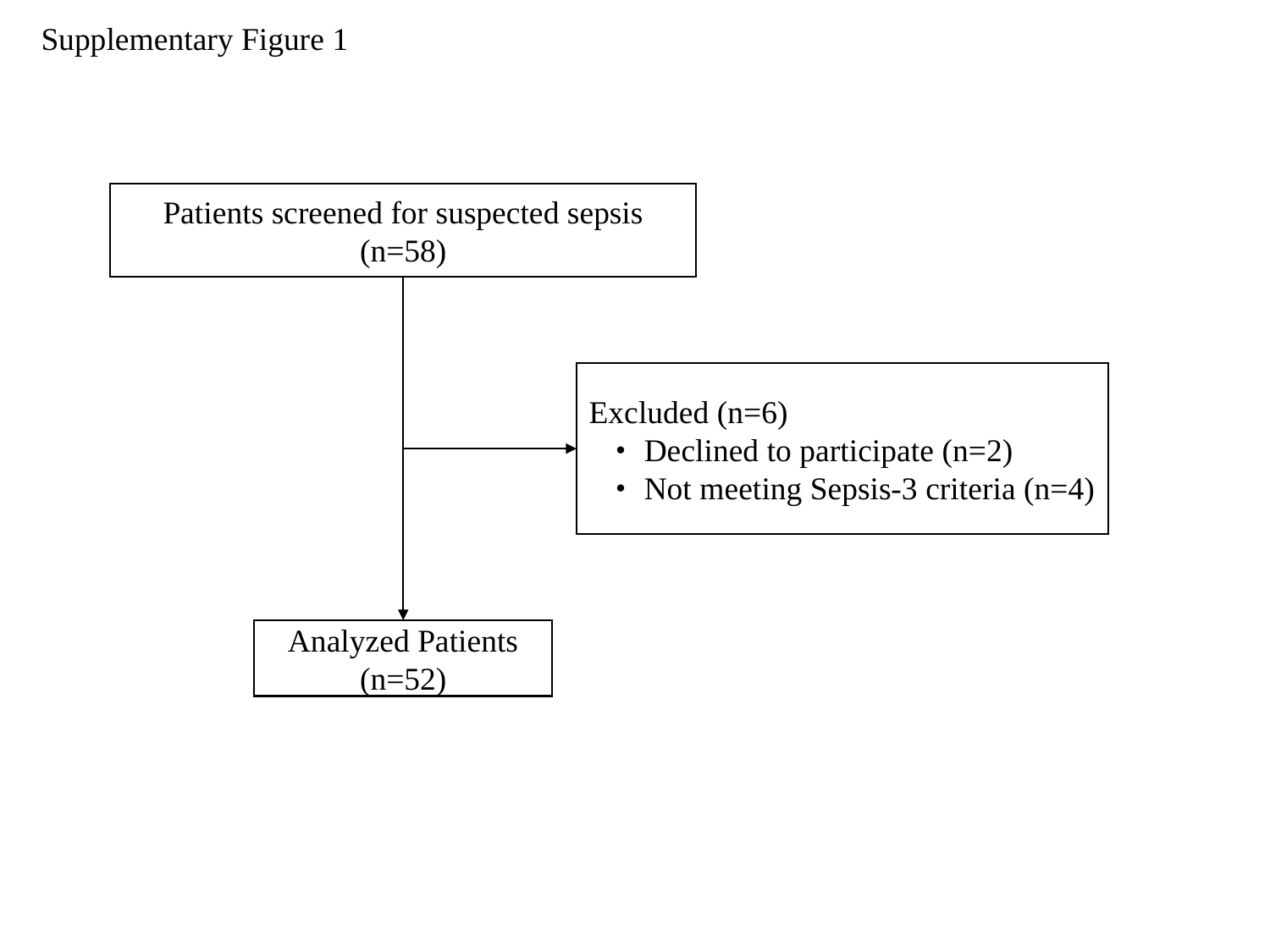

Supplementary Figure 1
Patients screened for suspected sepsis (n=58)
Excluded (n=6)
 ・Declined to participate (n=2)
 ・Not meeting Sepsis-3 criteria (n=4)
Analyzed Patients (n=52)
